# Osteosarcoma Cell-Derived Small Extracellular Vesicles Enhance Osteoclastogenesis and Bone Resorption Through Transferring MicroRNA-19a-3p

**DOI:** 10.3389/fonc.2021.618662

**Published:** 2021-03-25

**Authors:** Tingting Luo, Xiaocheng Zhou, Erhui Jiang, Lin Wang, Yaoting Ji, Zhengjun Shang

**Affiliations:** ^1^The State Key Laboratory Breeding Base of Basic Science of Stomatology (Hubei-MOST) & Key Laboratory of Oral Biomedicine Ministry of Education, School & Hospital of Stomatology, Wuhan University, Wuhan, China; ^2^Department of Oral and Maxillofacial Surgery, School & Hospital of Stomatology, Wuhan University, Wuhan, China; ^3^Department of Oral and Maxillofacial-Head and Neck Oncology, School and Hospital of Stomatology, Wuhan University, Wuhan, China

**Keywords:** osteosarcoma, small extracellular vesicles, osteoclast, miR-19a-3p, PTEN/PI3K/AKT signaling pathway

## Abstract

Osteosarcoma (OS) is the most common primary bone cancer characterized by an aggressive phenotype with bone destruction. The prognosis of OS patients remains unoptimistic with the current treatment strategy. Recently, osteoclasts are believed to play a crucial role in cancer bone metastasis. Thus, osteoclast could be a target both in bone destruction and cancer progression in OS. However, mechanisms governing osteoclastogenesis in OS remain poorly understood. miRNA delivered by small extracellular vesicles (sEVs) could mediate cellular communications. In this study, we investigated the effects of sEVs on osteoclastogenesis and osteoclast function, also clarified the underlying mechanism. We herein found that sEVs promoted pre-osteoclast migration, osteoclastogenesis and resorption by exposing RAW264.7 cells to sEVs derived from OS cells. Bioinformatics analysis showed that phosphatase tension homologue (PTEN), and miR-19a-3p were involved in OS progression. Overexpression of miR-19a-3p or sEVs’ miR-19a-3p promoted osteoclast formation and function through PTEN/PI3K/AKT signaling pathway, while inhibition of miR-19a-3p showed the contrary results. The bone marrow macrophages (BMMs) were used to verify the results. OS mice, which were established by subcutaneous injection of OS cells, exhibited increased levels of sEVs’ miR-19a-3p in blood. Moreover, micro-computed tomography (CT) and histomorphometry analysis demonstrated that OS mice exhibited osteopenia with increased number of osteoclasts. In conclusion, miR-19a-3p delivery *via* OS cell-derived sEVs promotes osteoclast differentiation and bone destruction through PTEN/phosphatidylinositol 3 -kinase (PI3K)/protein kinase B (AKT) signaling pathway. These findings highlight sEVs packaging of miR-19a-3p as a potential target for prevention and treatment of bone destruction and cancer progression in OS patients. And this finding provides a novel potentially therapeutic target for the bone metastasis.

## Introduction

Osteosarcoma (OS) is the most common primary bone malignancy with extremely poor prognosis and mortality. Patients may present pathologic fracture and bone metastasis. The combination of surgery, chemotherapy and adjuvant chemotherapy is current treatment strategy (1). Despite the significant progress that has been made in the diagnosis and treatment of OS during the last 30 years, the prognosis of OS patients remains unoptimistic (1–3). Thus, it is urgent to explore new targets for OS therapies (4). It is well established that osteoclasts are responsible for bone destruction in cancer bone metastasis such as breast cancer and prostate cancer and this is necessary for tumor expansion within the bone (5–7). And in OS progression, osteoclasts are believed to play a crucial role. It was reported that the receptor activator of nuclear factor kappa-β ligand (RANKL)/the receptor activator of nuclear factor kappa-β (RANK) signaling pathway plays an indispensable role in osteoclast differentiation and the MOTO-RANK^-/- OC^ mice exhibited delayed tumor initiation, prolonged life span, and fewer metastatic nodules in lung (8). Similarly, Lamoureux F reported that the osteoprotegerin (OPG), an inhibitor of osteoclast differentiation and function, inhibited osteolysis associated with OS and indirectly inhibited tumor progression indicating the significant contribution of osteoclasts in OS (9). Due to the crucial role both in bone resorption and OS progression, osteoclast may be a promising target for OS treatment and extensive research is required to further elucidate the effects of OS cells on osteoclast formation, activation and the underlying mechanism (8–10). However, the process of how OS cells affect osteoclast differentiation and function remains incompletely understood.

Small extracellular vesicles (sEVs), with typical marker proteins such as Reagents Heat shock protein 90 (Hsp90), tumor susceptibility gene 101 (Tsg101) and CD63 without calnexin, are the well-studied subpopulation (11, 12). It has been recognized that sEVs could mediate the communication between cells by transferring the specific material such as cytokine, signal molecular and miRNA (13, 14). Besides, the crucial role of sEVs as tools in monitoring tumor progression has attracted extensive attention (15). miRNAs delivered by sEVs has been regarded as potent mediators on tumor progression. The roles of miRNAs in OS have been demonstrated with increasing clinical implication. For instance, miR-19 has been identified to be overexpressed in various cancers including OS and plays a crucial role in tumor growth and progression (16–18). Similarly, it was reported that sEVs’ miR-25-3p and miR-675, highly expression in OS, affect the tumor progression and the circulating sEVs’ miRNA could be a novel diagnostic and prognostic biomarker (19, 20). Although various functions of sEVs’ miRNAs have been well identified in the biological behavior of OS cells, the role of these miRNAs in the osteoclast differentiation and function remains poorly understood.

Here, we reported that OS cells secret sEVs containing miRNAs that can be taken up into pre-osteoclasts, leading to osteoclastogenesis and bone resorption. Besides, treatment with sEVs leads to a decrease of the phosphatase tension homologue (PTEN) expression in osteoclasts. miR-19a-3p is highly expression in OS and promotes tumor initiation, progression and metastasis. Our study showed that miR-19a-3p is abundant in OS cells-secreted sEVs and that miR-19a-3p could promote osteoclastogenesis and function through PTEN/phosphatidylinositol 3 -kinase (PI3K)/protein kinase B (AKT) signaling pathway. Thus, sEVs packaging of miR-19a-3p could be a promising target for prevention and treatment of bone destruction and cancer progression in OS patients. And this finding provides a novel potentially therapeutic target for the bone metastasis.

## Materials and methods

### Materials and Mice

K7M2, MG63, HOS, RAW264.7 and human embryonic kidney 293 cells (293E cells) were purchased from China Center for Type Culture Collection (Shanghai, China). The female BALB/c nude mice were from Beijing Vital River Laboratory Animal Technology (Beijing, China). High-glucose Dulbecco’s modified eagle’s medium (DMEM) was from Hyclone (UT, USA). Fetal bovine serum (FBS) was obtained from Gibco (CA, USA). Cell Counting Kit-8 (CCK-8) was purchased from Dojindo (Kumamoto, Japan). Tartrate resistant acid phosphatase (TRAP) Kit and PKH26 were from Sigma-Aldrich (MO, USA). TRITC Phalloidin was from Yeasen (Shanghai, China). DAPI staining solution was from Beyotime (Shanghai, China). RANKL and macrophage-colony stimulating factor (M-CSF) were from R&D Systems (MN, USA). Primary antibodies for PTEN (Cat.No. 9559), AKT (Cat.No. 9272) and Phospho-AKT (p-AKT, ser473) (Cat.No. 4060) were purchased from Cell Signaling Technology (MA, USA). Primary antibody for Cathepsin K (CTSK) (Cat.No. AP7381) was from abcepta (Jiangsu, China). Primary antibodies for Hsp90 (Cat.No. 13171-1-AP), Tsg101 (Cat.No. 144971-AP), calnexin (66903-1-Ig) and matrix metalloproteinase-9 (MMP9) (Cat.No. 10375–2-AP) were from Proteintech (Hubei, China). CD63 (Cat.No. ab217345) was from abcam (MA, USA). BCA protein assay kit and CFSE were from Thermo Fisher Scientific (Rockford, USA). PCR related agents were from Takara (Tokyo, Japan).

### Isolation and Analysis of sEVs

K7M2, MG63 and HOS cells were cultured in DMEM containing 10% FBS at 37°C in a humidified 5% CO_2_ atmosphere. When 80% confluence was achieved, cells were washed with PBS and then cultivated with FBS-free DMEM. After 12 h, the supernatant was centrifuged at 800g, 10min; 1500g, 15min; 20000g for 35min and then sEVs were isolated by ultracentrifugation at 110000 g for 70 min. Following washed with PBS by using the same ultracentrifugation conditions, the sample was re-suspended in PBS.

Morphology of sEVs was observed with a transmission electron microscope (TEM) (HT7700, Japan). The particle size distribution of sEVs was analyzed with Nano-ZS ZEN 3600 (Malvern Instruments, UK). SEVs concentration was measured by BCA protein assay kit. For sEVs tracing, after incubation with 15μg/ml PKH26-labeled sEVs for 6 hours, RAW264.7 cells labeled by CFSE were washed with PBS and then observed with confocal microscope.

### Proliferation Viability and Migration Assays

RAW264.7 cells (5×10^4^/ml) were incubated in 96-well plates with or without sEVs for 1, 2, 3 and 4 days. After incubation with CCK-8 solution for 1 hour, optical density was determined at 450 nm.

RAW264.7 cells (1×10^4^ cells/ml) were pre-treated for 24 hours with sEVs (15μg/ml). Then cells were seeded into the upper chambers of 24-well transwell (8μ-pore filters) with 200μl FBS-free DMEM, whereas the lower chamber was filled with 500μl DMEM containing 10% FBS. Filters were fixed with PFA after 12 hours incubation. Cells that traversed to the reverse face were stained with crystal violet, photographed and counted.

### Preparation of Bone Marrow Macrophages (BMMs)

BMMs were isolated from femur and tibia marrow of 5 weeks old C57BL/6 female mice. Briefly, bone marrow cells were flushed from the femur and tibia with DMEM. After 24 hours incubation, non-adherent cells were collected and slowly layered on Ficoll-Hypaque gradient and centrifuged at 440g for 30 minutes at 4°C. Cells at the gradient interface were classified as BMMs. BMMs were cultured in DMEM containing 10% FBS and 30ng/ml M-CSF at 37°C in a humidified 5% CO2.

### Osteoclast Differentiation Assay and Fibrous Actin (F-actin) Ring

RAW264.7 cells (1×10^4^/ml) and BMMs (1×10^5^/ml) were seeded into 96-well plate with DMEM containing 10% FBS and 50ng/ml RANKL. 30ng/ml M-CSF was used for BMMs growth. SEVs^oligos^ from OS cells which were transfected with miR-19a-3p oligos including miR-19a-3p mimics-NC, mimics, inhibitor-NC and inhibitor (GenePharma, Jiangsu, China) were incubated with cells respectively. Every 2 days, the medium was replenished and osteoclasts were formed at 4-6 days. Then cells were stained with TRAP kit or TRITC Phalloidin according to instruction.

### Resorption Pit Assay

Calcium phosphate cements have been identified previously as synthetic biomimetic materials to investigate the osteoclast function (21, 22). Firstly, 96-well plates were incubated by simulated body fluid (SBF) containing 50% tris buffer (50nM, PH=7.4), 25% calcium stock solution (25mM CaCl_2_, 1.37M NaCl, 15 mM MgCl_2_ · 6H_2_O) and 25% phosphate stock solution (11.1 mM Na_2_HPO_4_, 42 mM NaHCO_3_) for 3 days with daily refreshment. Secondly, Calcium phosphate solution (CPS) was prepared by mixing 2.25 mM Na_2_HPO_4_, 4 mM CaCl_2_ and 0.14M NaCl in tris buffer. CPS was added to 96-well plates for 1day. Finally, plates were washed, dried at 37°C and sterilized with ultraviolet for 1 hour. Prior to use, plates were incubated with 100μl FBS for 1 hour.

RAW264.7 cells and BMMs were induced in plates above. After 7 days, plates were treated with 1 M sodium chloride containing 0.5% Triton100 to remove cells. Images were recorded and the relative area of pits was quantified by Image-Pro Plus 6.0 (Media Cybernetic, USA).

### Microarray Analysis

OS transcriptome microarray data (GSE87624) obtained from Gene Expression Omnibus (GEO) were utilized to identify the differentially expressed genes (DEGs) related to OS with Limma package of R language. The Gene oncology (GO) enrichment analysis was conducted with DAVID 6.8 to predict the potential functions and enrichment degree of DEGs in biological processes (BP), cellular components (CC) and molecular functions (MF). Additionally, the genes associated with tumorigenesis and progression in OS were analyzed by the DisGeNET database. Protein–protein interaction (PPI) network among DEGs was performed through STRING database. The database of miRDB, miRtarbase, RegRNA2.0 and TargetScan were used to predict the miRNAs that target PTEN.

### Dual-Luciferase Reporter Gene Assay

PTEN plasmids (pGL3) encoding wild or mutant 3′UTR were co-transfected with miR-19a-3p oligos into 293E cells. Highgene transfection reagent (abclone, Hubei, China) was used as the transfectant. After 48 hours transfection, luciferase activity was measured by Dual-Luciferase Reporter Assay Kit (Promega, Madison, USA). The sequence of synthesized oligonucleotides was listed (**Table 1**).

**Table 1:** Sequences of the synthesized oligonucleotides.

Oligonucleotides	Sequences	
	Sense (5’ to 3’)	Antisense (5’ to 3’)
miR-19a-3p mimics-NC	UUCUCCGAACGUGUCACGUTT	ACGUGACACGUUCGGAGAATT
miR-19a-3p mimics	UGUGCAAAUCUAUGCAAAACUGA	AGUUUUGCAUAGAUUUGCACAUU
miR-19a-3p inhibitor-NC	CAGUACUUUUGUGUAGUACAA	
miR-19a-3p inhibitor	UCAGUUUUGCAUAGAUUUGCACA	

### Quantitative Real-Time PCR Analysis

Total RNA was extracted by Trizol reagent. mRNA and miRNA were reverse transcribed with the PrimeScript™RT reagent Kit with gDNA Eraser and miRNA first strand cDNA synthesis (Sangon Biotech, Shanghai, China) respectively. The primer sequences were designed and synthetized (**Table 2**). Quantitative real-time PCR (qRT-PCR) was conducted with SYBR Premix Ex Taq™ II. Glyceraldehyde-3-phosphate dehydrogenase (GAPDH) or U6 was used to respectively normalize the mRNA expression or miRNA expression. The data were compared to normalized control values.

**Table 2:** Sequences of quantitative PCR primers.

Genes	Forward primer	Reverse primer
miR-466q	CGGTGCACACACACACATACGT	Universal PCR Primer R was purchased from Sangon Biotech (Shanghai, China)
miR-301a-3p	ACGGCAGTGCAATAGTATTGTCAAAGC	
miR-19b-3p	ACGTGTGCAAATCCATGCAAAACTGA	
miR-19a-3p	CGGCTGTGCAAATCTATGCAAAACTGA	
U6	Universal U6 Primer F was purchased from Sangon Biotech (Shanghai, China)	
PTEN	TCAGTTTGTGGTCTGCCAGC	GGCAATGGCTGAGGGAACTC
MMP-9	CAAAGACCTGAAAACCTCCAAC	GACTGCTTCTCTCCCATCATC
cathepsin K	GCTTGGCATCTTTCCAGTTTTA	CAACACTGCATGGTTCACATTA
GAPDH	GAPDH primer F and R were purchased from Sangon Biotech (Shanghai, China)	

### Western Blot Assay

Cells or sEVs were lysed with radioimmunoprecipitation lysis buffer containing protease and phosphatase inhibitor on ice. The protein concentration was measured by BCA assay. Western blot analyses were performed using 10% SDS-PAGE and 0.45μm polyvinylidene fluoride membranes. The membranes were incubated with primary antibody overnight at 4°C. Following incubating with anti-mouse/rabbit secondary antibody, the signals were detected with chemiluminescence (Bio-Rad, Singapore). The results were normalized to β-actin level.

### Tumorigenicity Assay in Nude Mice

The study was approved by the Ethics Committee of the Hospital of Stomatology at Wuhan University (approval numbers: S07918110A). PBS or K7M2 cells (5×10^6^) in 100μL PBS was injected subcutaneously into the right flank of the female BALB/c nude mice (n=5/group). All mice were raised in a SPF animal laboratory and randomly grouped. After 6 weeks, the blood was collected from the heart, followed the sEVs were extracted. After micro-CT analysis, the femurs and tibias were sectioned for Hematoxylin and Eosin (HE) and TRAP staining according to instructions.

### Micro–computed Tomography (CT) Scanning and Analysis

Femurs were scanned using a Skyscan 1176 micro-CT instrument (Broker, Kontich, Belgium) at a voxel size of 9μm. The volume of interest was above 0.5 mm from the growth plate of distal femur and the region of interest (ROI) in trabecular bone was manually defined as a constant threshold (50-100). subsequently, parameters within ROI were evaluated including bone volume per tissue volume (BV/TV), trabecular number (Tb. N), connectivity density (Conn. Dn), and bone surface (BS).

### Statistical Analyses

The independent experiments were performed in triplicate. Results were analyzed by SPSS software (SPSS, Chicago, USA). All quantitative data were presented as mean ± SEM from triplicates of independent experiments. Statistical comparisons were performed by student’s t-tests if the data is normally distributed (The normality test was (alpha=0.05) conducted with Shapiro-Wilk test) and the non-parametric should be used in other cases. Differences with values were considered statistically significant when P<0.05.

## Results

### OS Cell-secreted sEVs Promote Osteoclast Differentiation and Function

**Figure 1:**
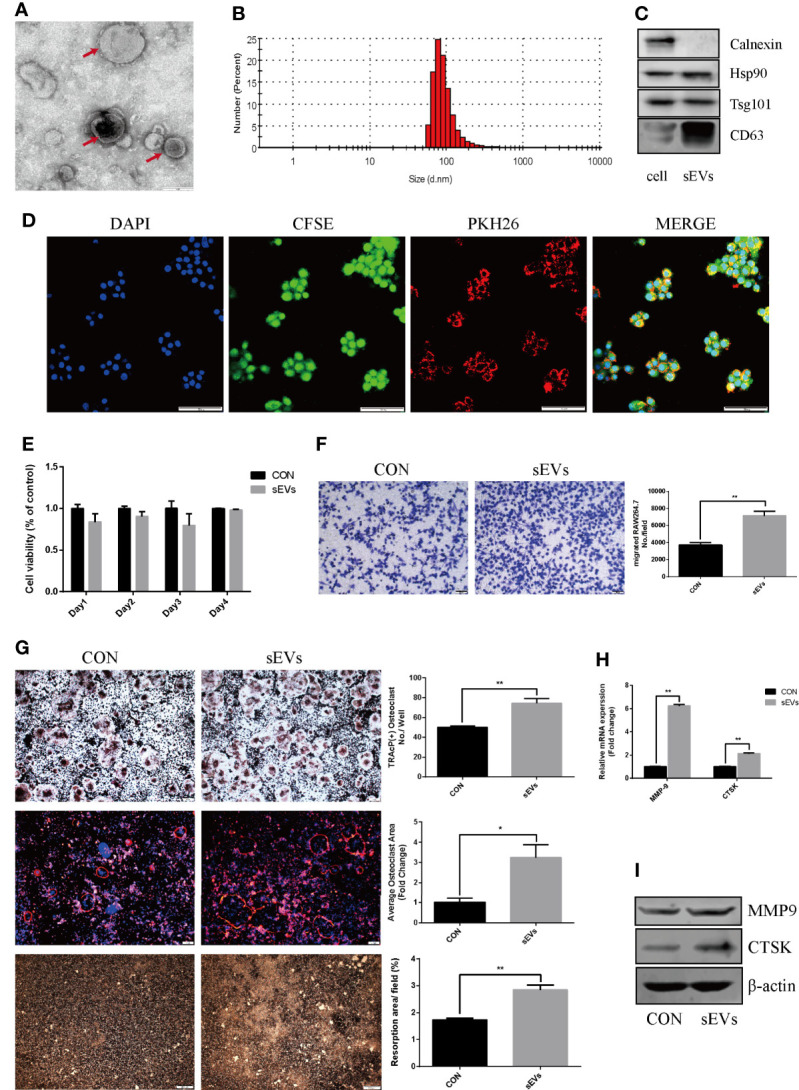
OS cell-secreted sEVs promote osteoclast differentiation and function. **(A, B)** SEVs released by OS cells were identified by using TEM (red arrow) and dynamic light scattering analysis. **(C)** Western blot analysis of markers: Calnexin, Hsp90, Tsg101, and CD63. **(D)** The uptake of PKH26-labeled sEVs by CFSE-labeled RAW264.7 cells observed under confocal microscope. **(E)** Proliferation viability of RAW264.7 cells detected by CCK-8 at 1, 2, 3 and 4 days in the presence or absence of sEVs. Optical density was measured at 450 nm. **(F)** Transwell migration assays of RAW264.7 cells untreated or pretreated for 24 hours with 15μg/ml of sEVs. **(G)** Representative images showing the osteoclastogenesis stained with TRAP kit, podosome belts stained with TRITC Phalloidin kit and hydroxyapatite resorption studied with resorption pit assay in each group and corresponding statistics. During the osteoclastic formation, RAW264.7 cells were treated with 15μg/ml of sEVs. After 4 days induction, the cells were TRAP and TRITC Phalloidin stained, and after 7 days, resorption pits were detected. **(H, I)** Representative qRT-PCR and Western Blot results of the effects of K7M2 cells’ sEVs on MMP-9 and CTSK expression. RAW264.7 cells were stimulated with RANKL (50 ng/mL) in the absence or presence of sEVs for 4 days before RNA and protein collection. Values are mean ± SEM of three independent experiments. *P < 0.05, **P < 0.01.

The sEVs from OS cells were purified by differential centrifugation and verified. After nanovesicles negative staining, sEVs (red arrow) exhibited as a typical cup-shaped morphology by TEM (**Figure 1A**). According to MISEV2018, EV subtypes smaller than 200 nm are defined as sEVs (12). In our study, the nanovesicles distribution was within 200 nm, which was consistent with the size range of sEVs (**Figure 1B**). The marker proteins including Hsp90, Tsg 101 and CD63 were expressed in these nanovesicles and cell lysis, whereas calnexin was only expressed in cell lysis (**Figure 1C**). SEVs tracing assay showed that the red fluorescence was observed in cytoplasm, indicating the internalization of sEVs in RAW264.7 cells (**Figure 1D**).

CCK8 assay showed that there was no obvious distinction between groups (**Figure 1E**). Migration assay revealed that pretreatment of K7M2 cells’ sEVs increased the migratory attitudes of RAW264.7 cells (The number of migrated RAW264.7 cell: CON vs sMVs=3704 ± 127.6 vs 7139 ± 246.9, p<0.01) (**Figure 1F**).

To determine the effect of sEVs on osteoclastogenesis and function, RAW264.7 cells were treated with sEVs from K7M2 cells. SEVs treatment significantly increased osteoclast number in RAW264.7 cells (**Figure 1G**). Rhodamine phalloidin staining exhibited the formation of well-defined podosome belts in mature osteoclasts, and larger osteoclasts with more nuclei were observed after sEVs treatment. Besides, treatment with sEVs statistically increased the resorption area and the expression of MMP-9, CTSK (**Figures 1H, I**).

### PTEN and miR-19a-3p are involved in OS

**Figure 2:**
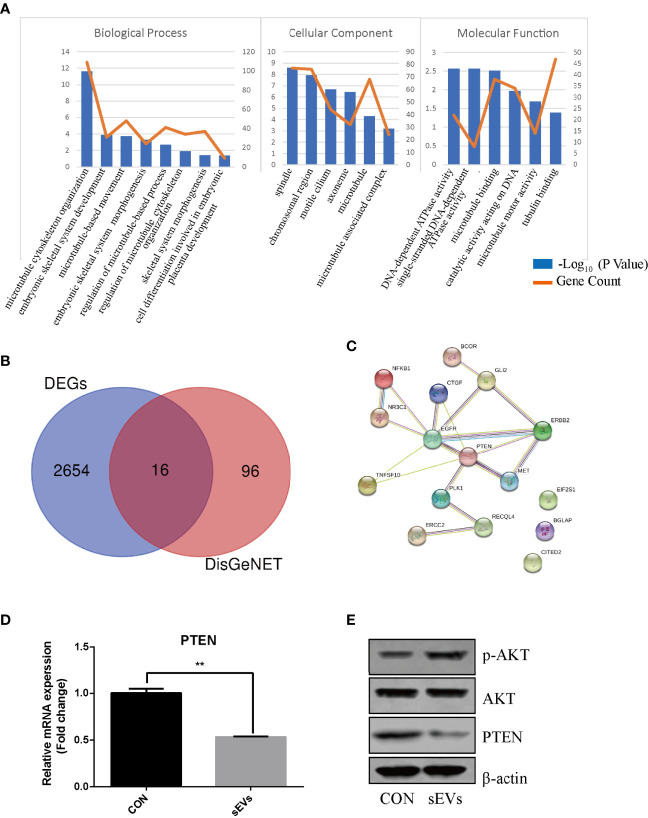
PTEN and miR-19a-3p are involved in OS. **(A)** GO enrichment analysis of DEGs in expression dataset of OS (GSE87624). The representative terms in BP, CC and MF were listed. X-axis represents the GO term. The left Y-axis represents the -Log_10_ (P Value) and the right Y-axis represents the count of genes. **(B)** The intersection of DEGs and the genes related to OS. The data DEGs was obtained from GSE87624 and the genes related to OS were from DisGeNET. **(C)** Interaction network among predicted OS-related DEGs. **(D)** QRT-PCR analysis of PTEN expression. **(E)** Western Blot analysis of the effects sEVs on p-AKT, AKT and PTEN expression. **(D, E)** were performed with RAW264.7 cells stimulated with RANKL in the absence or presence of K7M2 cells’ sEVs for 4 days. Values are mean ± SEM of three independent experiments. **P < 0.01.

The expression dataset of OS (GSE87624) was downloaded and analyzed in order to obtain DEGs in OS. There were 2670 DEGs in total. As shown in **Figure 2A**, GO enrichment analysis resulted variedly from GO classification and expression change of DEGs. As to BP, CC, MF, the DEGs markedly enriched in multiple terms and some of them are associated with bone metabolic process which is regulated by osteoblastic bone formation and osteoclastic bone resorption such as skeletal system morphogenesis, microtubule cytoskeleton organization, cell differentiation and energy metabolism. Therefore, it was proposed that DEGs in OS may participate in osteoclast formation and activation. To investigate the molecules associated with osteoclastogenesis in OS, the known genes related to OS (score > 0.05) from DisGeNET were intersected with DEGs, which confirmed 16 intersected genes (**Figure 2B**). PPI network showed a core position of PTEN (**Figure 2C**). Then, K7M2 cells’ sEVs treatment resulted in decreasing the expression of PTEN and activating AKT phosphorylation during the osteoclastogenesis (**Figures 2D, E**).

### miR-19a-3p Targets 3’UTR of PTEN

**Figure 3:**
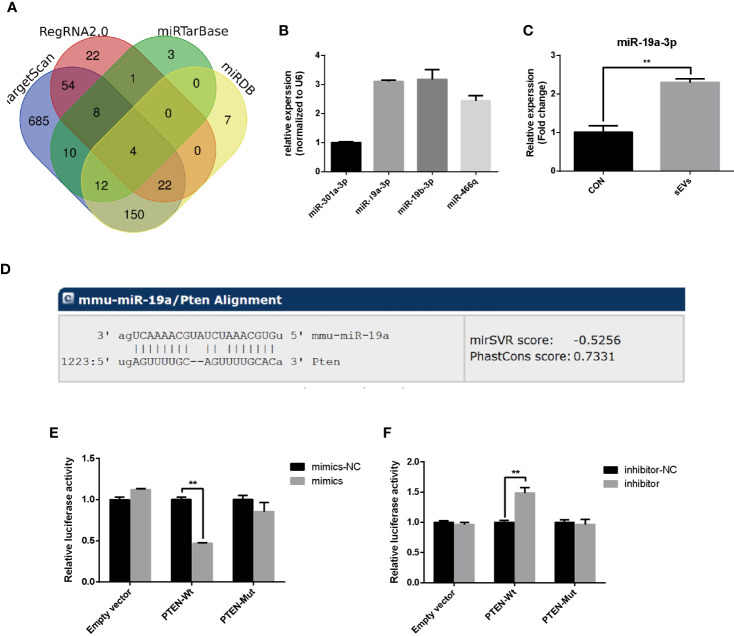
miR-19a-3p targets 3’UTR of PTEN. **(A)** Putative miRNAs that target the PTEN. The bioinformatics analysis was applied based on miRDB, miRtarbase, RegRNA2.0 and TargetScan. **(B)** The expression of predicted miRNA in sEVs derived from K7M2 cells determined by qRT-PCR. **(C)** qRT-PCR analysis of the effect of sEVs on miR-19a-3p expression after 4 days induction of RAW264.7 cells. **(D)** The miR-19a-3p binding site in PTEN 3’UTR. **(E, F)** Analysis of luciferase activity. 293E cells were co-transfected with the miR-19a-3p oligos and the wild-type or mutated type 3’UTR of PTEN, and luciferase activity was detected after 48 hours. Values are mean ± SEM of three independent experiments. **P < 0.01.

To predict miRNAs that target the PTEN, the bioinformatics analysis was applied based on databases including miRDB, miRtarbase, RegRNA2.0 and TargetScan and the results revealed that the PTEN gene was targeted by four miRNAs including miR-301a-3p, miR-19a-3p, miR-19b-3p and miR-466q (**Figure 3A**). miR-19a-3p is overexpressed in OS and there were some reports about the function of miR-19a-3p in the biological behavior of OS cells (15). However, the role of miR-19a-3p in osteoclastogenesis remains poorly understood. In our study, qRT-PCR showed that the miR-19a-3p was abundant in OS cells (K7M2)-secreted sEVs (**Figure 3B**). Treatment with sEVs increased the expression of miR-19a-3p during the osteoclastogenesis (**Figure 3C**). As shown in **Figure 3D**, PTEN 3’UTR contained potential miR-19a-3p binding sites. Dual-luciferase reporter assay revealed that the activity of firefly luciferase was significantly suppressed in 293E cells co-transfected with the miR-19a-3p mimics and the wild-type 3’UTR of PTEN whereas co-transfection with miR-19a-3p inhibitor and wild-type 3’UTR of PTEN enhanced the luciferase activity (**Figures 3E, F**).

### miR-19a-3p Enhances Osteoclastogenesis and Activation Through PI3K/AKT Signaling Pathway

**Figure 4:**
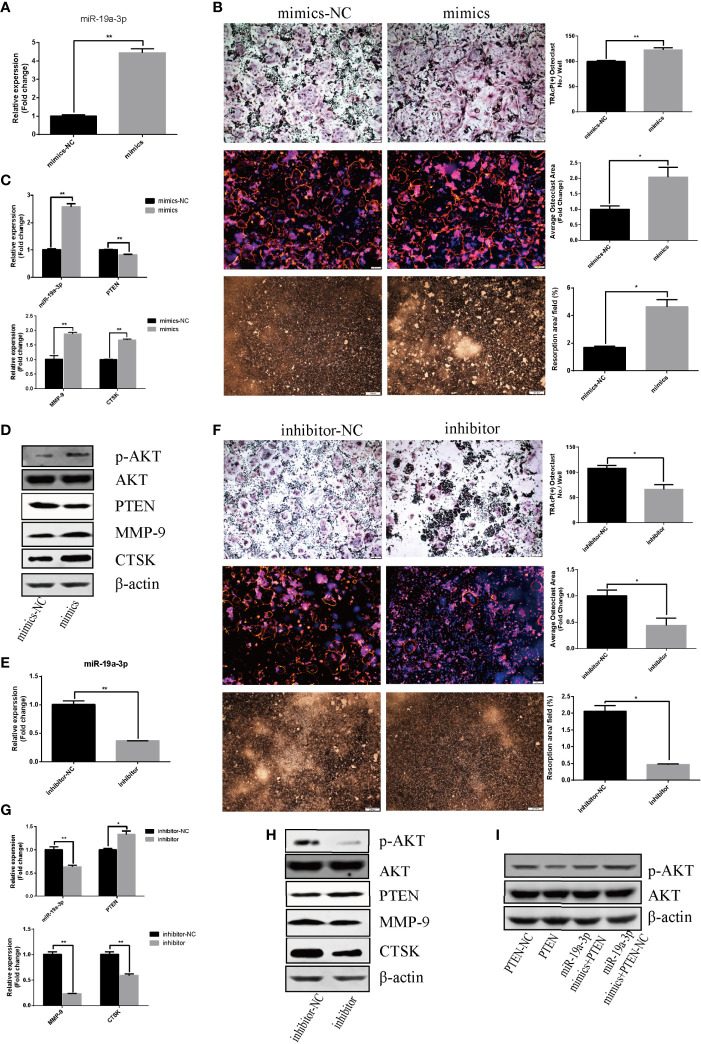
miR-19a-3p enhances osteoclastogenesis and activation through PI3K/AKT signaling pathway. **(A)** Transfection efficiency of miR-19a-3p mimics in RAW264.7 cells. miR-19a-3p expression was analyzed by qRT-PCR after 24 hours post transfection with mimics. **(B)** Representative images showing the osteoclastogenesis, podosome belts and hydroxyapatite resorption in each group and corresponding statistics. **(C, D)** Representative qRT-PCR and Western Blot analysis of miR-19a-3p, PTEN, MMP-9, CTSK, p-AKT and AKT expression. RAW264.7 cells transfected with miR-19a-3p mimic-NC or mimics were stimulated with RANKL (50 ng/mL) for 4 days before RNA and protein collection. **(E)** QRT-PCR analysis of miR-19a-3p level in RAW264.7 cells after 24 hours post transfection with inhibitor-NC or inhibitor. **(F)** Representative images showing the osteoclastogenesis, podosome belts and hydroxyapatite resorption in each group and corresponding statistics. **(G, H)** Representative qRT-PCR and Western Blot results of the effects of miR-19a-3p inhibitor on miR-19a-3p, PTEN, MMP-9, CTSK, p-AKT and AKT expression. RAW264.7 cells transfected with miR-19a-3p inhibitor-NC or inhibitor were stimulated with RANKL (50 ng/mL) for 4 days before RNA and protein collection. **(I)** AKT protein level and phosphorylation level in RAW264.7 cells transfected with PTEN-vector and/or miR-19a-3p mimics. Western Blot analysis was conducted after transfection 48 hours. Values are mean ± SEM of three independent experiments. *P < 0.05, **P < 0.01.

The effect of miR-19a-3p on osteoclast differentiation and activation was analyzed. The results indicated an augment of miR-19a-3p after 24 hours post transfection with mimics (**Figure 4A**). Overexpression of miR-19a-3p enhanced the number of osteoclasts, the size of podosome belt and the area of resorption (**Figure 4B**). After induction of RAW264.7 cells transfected with mimics, miR-19a-3p was high expression while PTEN was down-regulation (**Figure 4C**). As shown in **Figures 4C, D**, up-regulation of miR-19a-3p elevated the transcription and translation level of MMP-9, CTSK. Besides, PTEN protein level was reduced in the presence of miR-19a-3p mimics, whereas the p-AKT protein was increased (**Figure 4D**). In contract, miR-19a-3p was inhibited with the transfection of inhibitor after 24 hours (**Figure 4E**). After induction of RAW264.7 cells transfected with inhibitor, osteoclast number, podosome belt size and resorption area both were suppressed (**Figure 4F**). QRT-PCR revealed that miR-19a-3p was down-regulation while PTEN was up-regulation (**Figure 4G**). Simultaneously, the expression of MMP-9, CTSK were suppressed (**Figures 4G, H**). Besides, the protein level of PTEN was increased in the presence of miR-19a-3p inhibitor while the p-AKT was decreased (**Figure 4H**). As shown in **Figure 4I**, p-AKT was down-regulation in RAW264.7 cells transfected with PTEN. Notably, p-AKT was markedly decreased following the transfection of both miR-19a-3p and PTEN compared to transfection of miR-19a-3p alone. Base on the above results, miR-19a-3p could activate the PI3K/AKT signaling pathway by down-regulation of PTEN.

### SEVs’ miR-19a-3p Promotes Osteoclastogenesis and Activation

**Figure 5:**
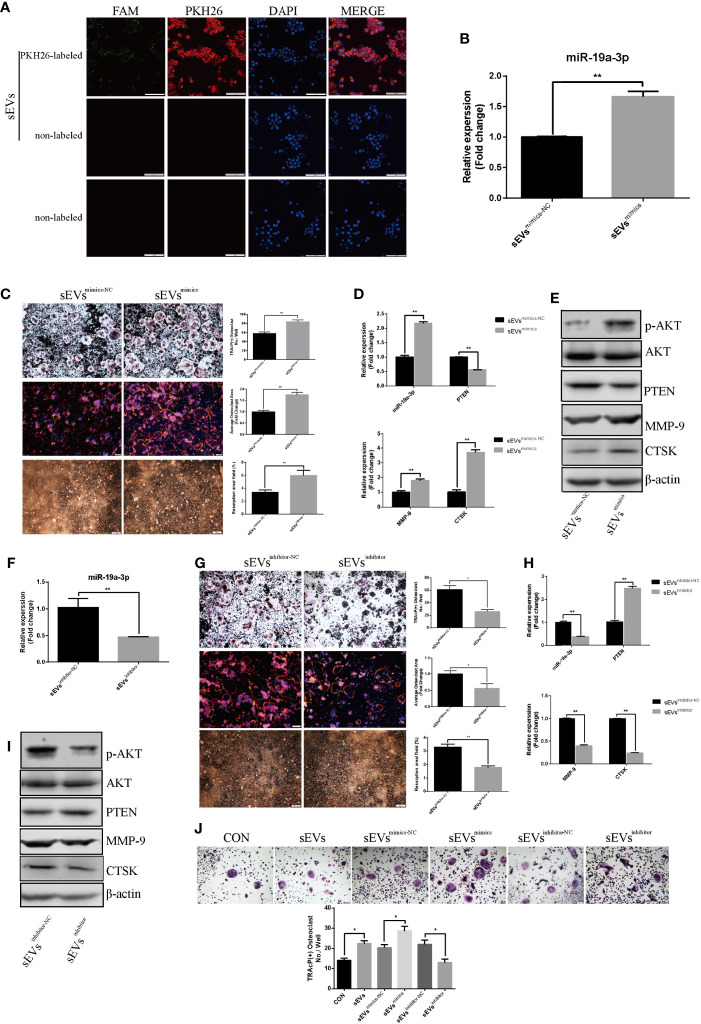
SEVs’ miR-19a-3p promotes osteoclastogenesis and activation. **(A)** uptake of sEVs from OS cells transfected with FAM-tagged miR-19a-3p. **(B)** QRT-PCR analysis of miR-19a-3p level in K7M2 cells’ sEVs^mimics-NC^ and sEVs^mimics^. **(C)** Representative images showing the osteoclastogenesis, podosome belts and hydroxyapatite resorption in each group and corresponding statistics. **(D, E)** Representative qRT-PCR and Western Blot results of the effects of K7M2 cells’ sEVs^mimcs^ on miR-19a-3p, PTEN, MMP-9, CTSK, p-AKT and AKT expression. RAW264.7 cells treated with K7M2 cells’ sEVs^mimics-NC^ or sEVs^mimics^ were stimulated with RANKL (50 ng/mL) for 4 days before RNA and protein collection. **(F)** QRT-PCR analysis of miR-19a-3p level in K7M2 cells’ sEVs^inhibitor-NC^ and sEVs^inhibitor^. **(G)** Representative images showing the osteoclastogenesis, podosome belts and hydroxyapatite resorption in each group and corresponding statistics. **(H, I)** Representative qRT-PCR and Western Blot results of the effects of K7M2 cells’ sEVs^inhibitor^ on miR-19a-3p, PTEN, MMP-9, CTSK, p-AKT and AKT expression. RAW264.7 cells treated with K7M2 cells’ sEVs^inhibitor-NC^ or sEVs^inhibitor^ were stimulated with RANKL (50 ng/mL) for 4 days before RNA and protein collection. **(J)** Representative images showing the osteoclastogenesis differentiated from BMMs in each group and corresponding statistics. Values are mean ± SEM of three independent experiments. *P < 0.05, **P < 0.01.

As shown in **Figure 5A**, sEVs from OS cells transfected with FAM-tagged miR-19a-3p mimics were further labeled with PKH26 and then incubated with RAW264.7 cells. Both FAM and PKH26 fluorescence were observed around the nuclear. However, no FAM or PKH26 fluorescence was observed in cells treated with non-labeled sEVs or naked FAM-tagged miR-19a-3p. Thus, OS cell-secreted sEVs’ miR-19a-3p could be internalized by RAW264.7 cells.

QRT-PCR showed that miR-19a-3p was higher expression in K7M2 cells sEVs^mimics^ compared to sEVs^mimics-NC^ (**Figure 5B**). Treatment with sEVs^mimics^ efficiently potentiated the osteoclast number, podosome belt size and resorption area (**Figure 5C**). As shown in **Figure 5D**, miR-19a-3p was up-regulation while the PTEN was down-regulation upon the treatment with sEVs^mimics^. Meanwhile, the expression of MMP-9 and CTSK were increased (**Figures 5D, E**). PTEN protein was reduced after treatment with sEVs^mimics^, while p-AKT protein was increased (**Figure 5E**). Conversely, sEVs^inhibitor^ contained less miR-19a-3p (**Figure 5F**) and significantly decreased the osteoclast number, podosome belts size and resorption area (**Figure 5G**). The expression of MMP-9 and CTSK in transcription and translation level were decreased (**Figures 5H, I**). Besides, treatment with sEVs^inhibitor^ upregulated the expression of PTEN but downregulated the phosphorylation of AKT (**Figure 5I**). To confirm the effects of sEVs’ miR-19a-3p on osteoclast differentiation, similar experiments were conducted using another model of osteoclast generation: mouse BMMs (**Figure 5J**). Obviously, sEVs efficiently potentiated the formation of osteoclast and sEVs^mimics^ further potentiated the effect, whereas sEVs^inhibitor^ largely abrogated this promotional effect.

Furthermore, miR-19a-3p was abundant in MG63 and HOS cells-secreted sEVs (**Supplementary Figure A**). SEVs derived from MG63 and HOS cells efficiently increased the number of osteoclast and sEVs^mimics^ further potentiated the effect, whereas sEVs^inhibitor^ largely abrogated this promotional effect (**Supplementary Figures B, C**).

### The sEVs’ miR-19a-3p from OS Mice Results in Osteopenia *In Vivo*

**Figure 6:**
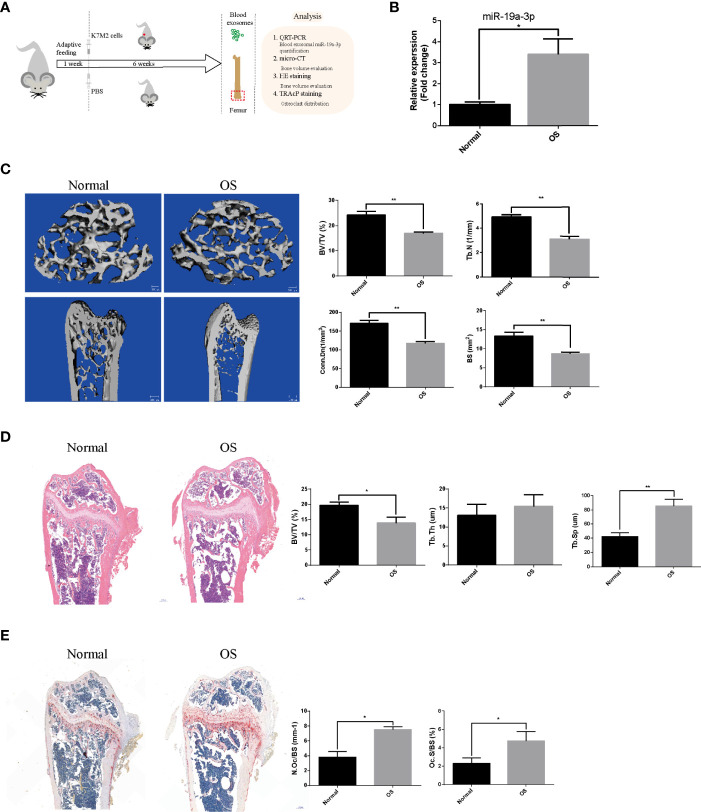
The sEVs’ miR-19a-3p from OS mice results in bone osteopenia *in vivo*. The mice were randomly divided into two groups (N=5 per group): Normal group and OS group. **(A)** Schematic illustration of the establishment of OS model and the experimental design. **(B)** Representative qRT-PCR analysis of the blood level of sEVs’ miR-19a-3p in normal and OS mice. **(C)** Representative micro-CT images showing the bone loss in OS femur and the analysis of parameters regarding bone microstructure, including bone volume per tissue volume (BV/TV), trabecular number (Tb. N), connectivity density (Conn. Dn), and bone surface (BS). **(D)** Representative images of HE staining of decalcified bone sections and quantitative analyses of BV/TV, Tb. Th and Tb. Sp in tissue sections. **(E)** Representative images of TRAP staining of decalcified bone sections and quantitative analyses of N.Oc/BS and Oc.S/BS in tissue sections. Values are mean ± SEM from five mice data per group. *P < 0.05, **P < 0.01.

The OS models were established by injecting subcutaneously K7M2 cells (**Figure 6A**). The blood level of sEVs’ miR-19a-3p in OS mice was evidently higher than control mice (**Figure 6B**). Micro-CT of femurs revealed osteopenia in OS mice (**Figure 6C**). Namely, Bone parameters including BV/TV, Tb. N, Conn. Dn, and BS were all lower in OS group (**Figure 6C**). Consistently, histological examination displayed the osteopenia in OS mice (**Figure 6D**). BV/TV were lower and Tb. Sp were larger in OS mice although there was no statistical difference in Tb. Th (**Figure 6D**). Furthermore, N.Oc/BS and Oc.S/BS in sections were analyzed and there was a marked increase in the number of osteoclast in OS mice (**Figure 6E**).

## Discussion

OS is clinically characterized by extensive bone destruction and metastasis in which osteoclasts play essential roles (23–26). It was reported that osteoclasts are responsible for bone destruction and metastasis in bone metastases (5). Herein, osteoclast could be a target both in bone destruction and cancer progression. OS, deriving from mesenchymal tissue, could produce RANKL to regulate osteoclast differentiation and activation (8). However, it is unclear whether there are additional approaches for the OS cell-osteoclast communication. SEVs, with enclosed lipid bilayer membrane, could stably transport and efficiently deliver biologically active molecules such as mRNAs, miRNAs to recipient cells. Raimondi L et al. reported that exosomes isolated from three OS cell lines induced osteoclast differentiation (27). Our results confirm that sEVs derived from OS cells could be absorbed by pre-osteoclasts further promoting the osteoclast differentiation and resorption. Whereas the related mechanism remains incompletely understood.

According to the bioinformatics analysis, PTEN was located at the core position among DEGs in OS. Consequently, PTEN may act as a crucial regulator in OS progression. It was reported that PTEN expression was negatively associated with OS metastasis and survival (28). Besides, Blüml S showed that PTEN ^(-/-)^ displayed increased osteoclast number and bone resorption, indicating the negative regulation of osteoclastogenesis and function by PTEN (29). Interestingly, our results exhibited a decrease of the PTEN expression in osteoclasts after treatment with sEVs. Therefore, we convinced that sEVs could promote osteoclast formation and function by delivering biologically active molecules into RAW264.7 cells and further down-regulating the expression of PTEN. Previous studies have suggested that PTEN can be directly targeted by multiple miRNAs such as miR-214, miR-142-5p, and affect the osteoclast differentiation and function (30, 31). miR-19a-3p is overexpressed in OS and there were some reports about the facilitating function of miR-19a-3p in the biological behavior of OS cells including proliferation, migration, invasion and metastasis (16). The negative role of miR-19a-3p in OS cells’ apoptosis was also reported (32). Zhang B et al. demonstrated that downregulation of miR-19a-3p enhanced the chemosensitivity of OS cells by elevating the expression of PTEN (33). Besides, it is well known that the overexpression of miR-19a-3p is an underlying risk of poor prognosis in many human malignancies, especially in osteosarcoma (34, 35). Elevated miR-19a-3p expression is associated with the potential of lymph node metastasis (34, 35). Moreover, recent study highlighted the positive role of miR-19a-3p in osteoblast differentiation by targeting Hoxa5 (36). However, the role of miR-19a-3p in osteoclastogenesis remains poorly understood. Our studies showed that miR-19a-3p promoted osteoclast differentiation and function by targeting and down-regulating PTEN. Herein, due to the promotion of miR-19a-3p in bone resorption and OS progression, miR-19a-3p may be a promising target for OS therapy.

Studies have proved that PTEN is a potent inhibitor of PI3K/AKT signaling cascade. Tian K et al. demonstrated that miR-23a plays a positive effect on migration and invasion through PI3K/AKT pathway *via* suppressing the expression of PTEN in OS (37). Consistently, our study showed that miR-19a-3p activated PI3K/AKT signaling pathway through suppressing the expression of PTEN. The above findings were further supported by the study reported by Adapala NS et al., which suggested that the activation of PI3K/AKT signaling pathway leads to the increase in osteoclast formation and resorption *in vitro* (38).

Furthermore, we also showed that miR-19a-3p was high expression in OS cell-derived sEVs and could be delivered into RAW264.7 cells by sEVs. Treatment with sEVs^mimics^ enhanced the osteoclast formation, resorption and the expressions of MMP-9, CTSK. Simultaneously, sEVs^mimics^ inhibited the expression of PTEN and increased the phosphorylation of AKT. However, these effects were inversed when RAW264.7 cells were treated with sEVs^inhibitor^. From the above results, we convinced that sEVs derived from OS cells could promote osteoclast differentiation and function *via* miR-19a-3p targeting PTEN/PI3K/AKT signaling pathway. Besides, the conclusion was confirmed by the model of BMMs.

Lim JS found that 47.5% patients had osteoporosis and 30.0% had osteopenia and the regions affected covered femur neck of OS site unaffected femur neck, lumbar spine, and total body (39). Besides, Holzer et al. reported that 65% OS patients who received chemotherapy had BMD deficits in 16 ± 2.2 years follow-up from diagnosis (40). In this study, we established OS mice model to investigate the effect of OS on osteoclastogenesis and the osteoclastic bone resorption by injecting subcutaneously K7M2 cells into the flank of nude mice excluding the direct effect of OS cells on bone microstructure. In our study, the blood sEVs’ miR-19a-3p was higher in OS mice than normal. Micro CT exhibited the osteopenia in OS mice and the histological examination showed the decrease in bone parameters and the increase in the number of osteoclasts in femur. Above all, we demonstrated that blood sEVs’ miR-19a-3p derived from OS could be considered as a crucial factor in osteoclastogenesis and bone destruction, and we clarified the associated mechanism *in vitro* study. However, we cannot precisely regulate the expression of sEVs’ miR-19a-3p in OS model to further verify the results due to the limitation of the current technology *in vivo* and this will be the focus of our future research.

Given the significant role of osteoclast in bone destruction and OS progression, sEVs’ miR-19a-3p becomes a potential target for the treatment of OS. Recently, it has emerged that osteoclasts play a crucial role in local OS growth and metastasis. Therefore, additional studies should be performed to explore the effect of osteoclasts on OS progression.

## Conclusion

**Figure 7:**
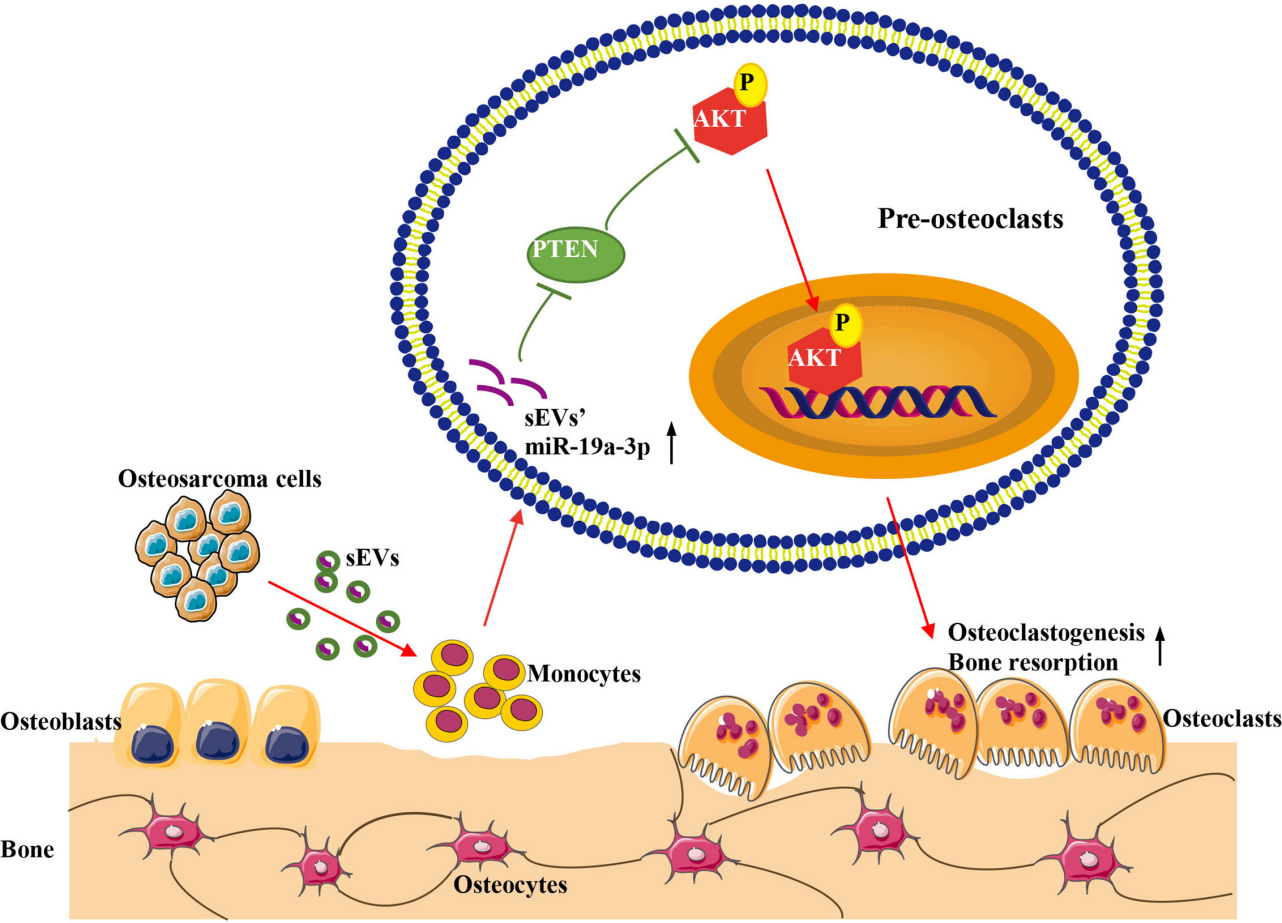
Schematic model for the promotion of sEVs derived from OS cells on osteoclast differentiation and function. OS cells deliver sEVs packaging miR-19a-3p into pre-osteoclasts. Subsequently, sEVs’ miR-19a-3p targets PTEN and down-regulates the expression of PTEN resulting in the activation of phosphorylation of AKT. Consequently, osteoclast differentiation and function are promoted.

In conclusion, our study demonstrated that OS cell-derived sEVs could deliver miR-19a-3p to promote osteoclast differentiation and function resulting in bone destruction through PTEN/PI3K/AKT signaling pathway (**Figure 7**). This finding provides a novel potentially therapeutic target against OS and may present a new target for the treatment of bone metastasis in cancers.

## Data Availability Statement

The raw data supporting the conclusions of this article will be made available by the authors, without undue reservation.

## Ethics Statement

The animal study was reviewed and approved by Ethics Committee of the Hospital of Stomatology at Wuhan University.

## Author Contributions

TL conducted the research, created the figures, and wrote the manuscript. XZ, EJ, and LW participated in the laboratory experiments and performed the data analysis. YJ and ZS revised the manuscript and supervised the research. All authors contributed to the article and approved the submitted version.

## Funding

This study was supported by the National Natural Science Foundation of China (Grant 81672666 to Z. Shang, Grant 81700772 to YJ).

## Conflict of Interest

The authors declare that the research was conducted in the absence of any commercial or financial relationships that could be construed as a potential conflict of interest.

## Supplementary Material

Supplementary Figure 1 SEVs’ miR-19a-3p derived from MG63 and HOS cells promotes osteoclastogenesis. **(A)** The expression of predicted miRNA in sEVs derived from MG63 and HOS cells determined by qRT-PCR. **(B)** Representative images showing the osteoclastogenesis after treatment with MG63 cells’ sEVs and corresponding statistics. **(C)** Representative images showing the osteoclastogenesis after treatment with HOS cells’ sEVs and corresponding statistics. *P<0.05, **P<0.01.
