# Supplementary figures and images for: Osteosarcoma Cell-Derived Small Extracellular Vesicles Enhance Osteoclastogenesis and Bone Resorption Through Transferring MicroRNA-19a-3p
Source: Front Oncol. 2021 Mar 25;11:618662. doi: 10.3389/fonc.2021.618662 (PMC8029976; doi:10.3389/fonc.2021.618662)

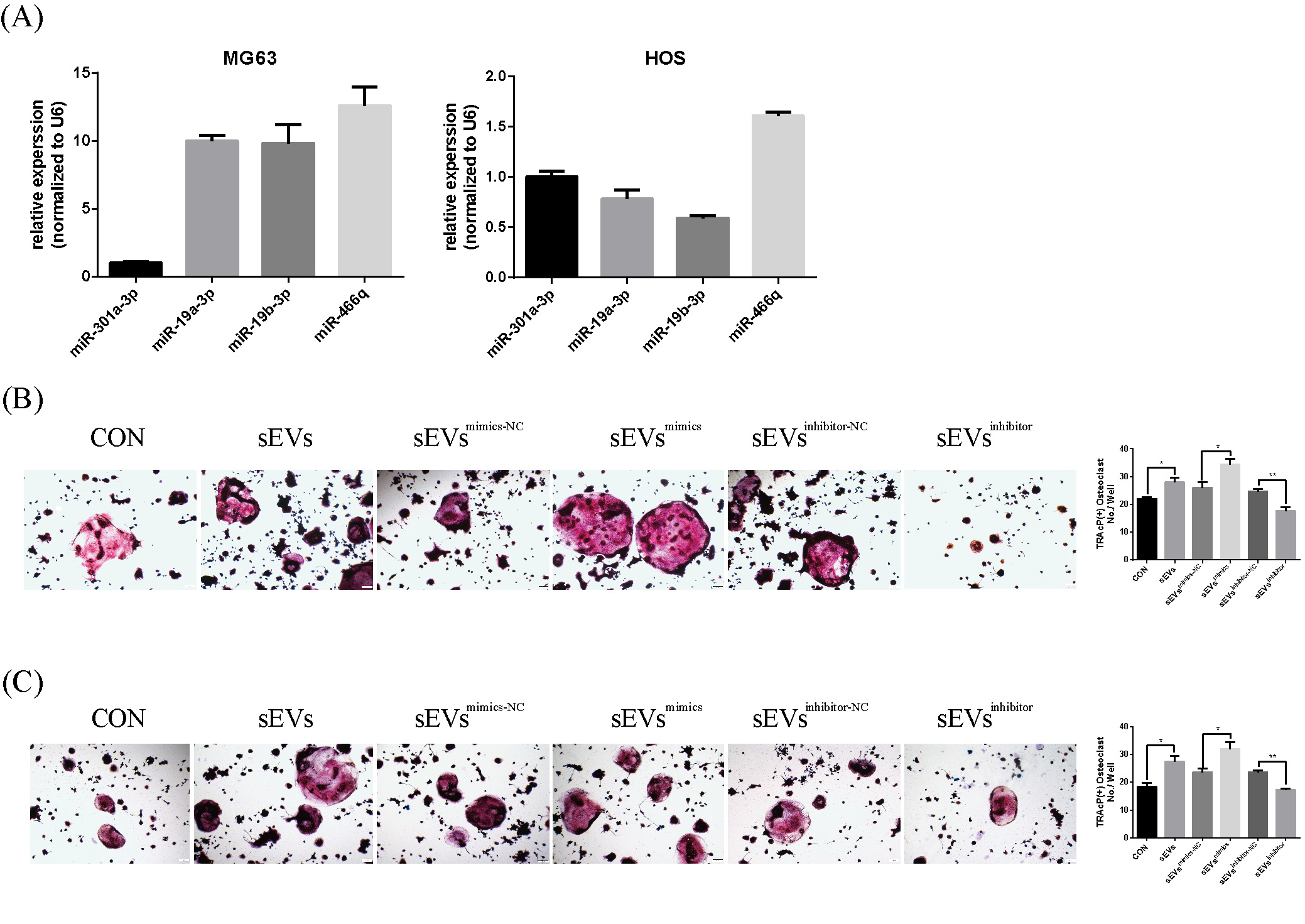

Supplement: Supplementary Figure 1 — SEVs’ miR-19a-3p derived from MG63 and HOS cells promotes osteoclastogenesis. (A) The expression of predicted miRNA in sEVs derived from MG63 and HOS cells determined by qRT-PCR. (B) Representative images showing the osteoclastogenesis after treatment with MG63 cells’ sEVs and corresponding statistics. (C) Representative images showing the osteoclastogenesis after treatment with HOS cells’ sEVs and corresponding statistics. *P<0.05, **P<0.01. [file Image_1.jpeg]
